# $T_{2}$‐Weighted Imaging of Water, Fat and Silicone

**DOI:** 10.1002/mrm.70253

**Published:** 2026-01-22

**Authors:** Aizada Nurdinova, Xuetong Zhou, Julio A. Oscanoa, Preya Shah, Kawin Setsompop, Bruce L. Daniel, Brian A. Hargreaves

**Affiliations:** ^1^ Department of Radiology Stanford University Stanford California USA; ^2^ Department of Bioengineering Stanford University Stanford California USA; ^3^ Department of Electrical Engineering Stanford University Stanford California USA

**Keywords:** chemical‐shift encoding, compressed sensing, joint multi‐echo reconstruction, Nyquist ghost correction, silicone breast imaging, $T_{2}$‐weighted contrast

## Abstract

**Purpose:**

Magnetic resonance imaging (MRI) is a sensitive method for assessing silicone implant integrity, with $T_{2}$‐weighted imaging being essential for detecting abnormalities in surrounding tissue. Silicone breast imaging protocols often require multiple tailored sequences for species suppression and diagnostic contrast. We propose a single sequence suitable for patients with or without implants that enables $T_{2}$‐weighted, high‐quality imaging and three‐species separation within a clinically feasible scan time.

**Methods:**

Our approach uses a 2D fast spin echo (FSE) sequence with seven bipolar multi‐echo gradient echo readouts, enabling field mapping and water–fat–silicone separation. Incoherent $k_{y}$–TE undersampling combined with joint multi‐echo reconstruction leverages temporal correlations and applies compressed sensing regularization directly to the separated species.

**Results:**

We achieve high‐resolution, artifact‐free water, fat, and silicone (WFS) images across three planes from one sequence, regardless of shim quality, and for different breast implant types. Compared to independent echo reconstruction and separation, joint multi‐echo reconstruction with incoherent $k_{y}$–TE sampling allows acceleration of R=6, reducing scan time to 2.5 minutes.

**Conclusion:**

We demonstrate a robust $T_{2}$‐weighted technique that provides reliable water–fat–silicone imaging in 2.5 minutes, enabling uniform breast protocols for patients with and without silicone implants.

## 1 Introduction

Breast augmentation and reconstruction are among the most common cosmetic surgery procedures. Around 84% of breast implants are silicone‐based, while 16% are saline [1]. Breast MRI, due to its absence of ionizing radiation, high resolution, and ability to depict water and silicone, is highly effective for detecting ruptures and evaluating complications related to silicone implants, usually without the use of contrast agents [2].

Typical MRI protocols for silicone implants use high‐resolution 2D or 3D $T_{1}$‐ and $T_{2}$‐weighted sequences plus multiplanar $T_{2}$ scans for water‐ and silicone‐specific imaging [2]. $T_{1}$‐weighted images support anatomical assessment and detection of $T_{1}$‐bright hematomas, while $T_{2}$‐weighted images depict cysts, ducts, lymph nodes, fibroadenomas, and certain malignancies [3]. Dark $T_{2}$ signal may indicate clips, biopsy markers, or invasive carcinoma. Water‐ and silicone‐specific $T_{2}$ imaging distinguishes normal findings (folds, reactive fluid) from rupture or granulomas, demonstrates abscess seromas and double‐lumen saline compartments, and identifies capsular contraction and free silicone in surrounding tissues.

At 3T, silicone has relaxation times of $T_{1}∼1\;s$ and $T_{2}∼130\;\text{ms}$ [4, 5], with a resonance frequency of −4.9ppm, close to fat at −3.4ppm [6]. This proximity complicates center frequency calibration and can cause artifacts with selective suppression, Dixon species swaps [7, 8], blurring in non‐Cartesian imaging, and shimming or RF‐selective imaging errors.

Silicone‐specific $T_{2}$‐weighted imaging methods are broadly classified as TR‐based or Dixon‐based. TR‐based techniques such as double inversion recovery (DIR) or combined water‐ and fat‐suppression [9, 10] often suffer from reduced SNR and suppression failures that may mimic rupture. Dixon‐based approaches offer higher SNR images [11, 12] but require robust field mapping. Recently, a monopolar multi‐echo GRE sequence enabled $T_{1}$‐weighted, high‐resolution water–fat–silicone imaging free of suppression artifacts, improving implant assessment [13, 14] Extending this to a $T_{2}$‐weighted sequence remains challenging due to prolonged echo spacing and the need for multiple shifted echoes, although a three‐echo 2D FSE method was proposed [15].

We present a $T_{2}$‐weighted multispecies breast imaging method that removes the need for tissue suppression and enables routine diagnostic imaging regardless of shim quality or breast implants. In cases with silicone implants, it also provides comprehensive implant imaging, which is especially useful when the implant type is unknown. The approach integrates a multi‐echo 2D FSE sequence with incoherent $k_{y}$–TE sampling and joint compressed sensing reconstruction to extract water, fat, silicone, and combined images with displacement correction. We evaluate undersampling strategies, compare the joint method to a two‐stage reconstruction demonstrating accelerations up to R=6, and present images relative to the STIR two‐echo Dixon 3D FSE reference.

## 2 Methods

### 2.1 Background

#### 2.1.1 Water, Fat, and Silicone (WFS) Separation

Chemical‐shift‐encoded (CSE) MRI samples signals at multiple echo times, encoding species‐specific resonant frequencies that are subsequently resolved by fitting the data to a spectral model [7, 16, 17]. For breast imaging in the presence of silicone implants, we aim to separate WFS components from multi‐echo images [13]. Specifically, assuming a nine‐peak fat [18] and a single‐peak silicone models, and given $N_{t}$ echo images, the CSE system can be expressed in matrix form:

(1) $$\underset{x}{\underbrace{\left(\begin{array}{l} x(t_{1})\\ \vdots \\ x(t_{N_{t}}) \end{array}\right)}}=\underset{A(\mu )}{\underbrace{\left(\begin{array}{lll} e^{\mu t_{1}} & \Phi_{f}(t_{1})e^{\mu t_{1}} & \Phi_{s}(t_{1})e^{\mu t_{1}}\\ \vdots & \vdots & \vdots \\ e^{\mu t_{N_{t}}} & \Phi_{f}(t_{N_{t}})e^{\mu t_{N_{t}}} & \Phi_{s}(t_{N_{t}})e^{\mu t_{N_{t}}} \end{array}\right)}}\;\underset{\rho }{\underbrace{\left(\begin{array}{l} \rho_{w}\\ \rho_{f}\\ \rho_{s} \end{array}\right)}}$$

where $x(t_{j})$ is the j‐th gradient echo image, $t_{j}={\text{TE}}_{j}$ is the j‐th gradient echo time with respect to the spin echo, $\rho_{w}$, $\rho_{f}$ and $\rho_{s}$ are the complex WFS images of sizes $N_{\text{pixels}}\times 1$, while $\mu =2\pi if_{B}-R_{2}^{\prime }$ combines the off‐resonance $f_{B}$ map and the effective transverse relaxation rate $R_{2}^{\prime }\;=R_{2}^{*}-R_{2}$ [8] map and is also of shape $N_{\text{pixels}}\times 1$. The fat and silicone phasors are pixel‐independent and defined as:

(2) $$\Phi_{f}(t_{j})=\overset{N_{f}}{\underset{p=1}{\sum }}\alpha_{p}e^{2\pi if_{f,p}t_{j}},\;\Phi_{s}(t_{j})=e^{2\pi if_{s}t_{j}},$$

where $f_{s}$ is the silicone off‐resonance frequency with respect to the water peak, $f_{f,p}$ and $\alpha_{p}$ are the fat peak frequencies and relative amplitudes, satisfying $\sum_{p=1}^{N_{f}}\alpha_{p}=1$, and $N_{f}=9$ is the number of fat peaks [18, 19, 20, 21]. Thus, x has dimensions of $N_{\text{pixels}}\times N_{t}$, and ρ of $N_{\text{pixels}}\times N_{\text{species}}$, and A(μ) has a block‐diagonal form across pixels and maps ρ↦x.

The estimation of the field inhomogeneity map $f_{B}$ and species separation is a nonlinear, nonconvex problem. For imaging with significant field‐map variations, this can be robustly solved using graph‐cut algorithms that achieve global convergence within the search range [13, 22, 23, 24, 25]. Spatial smoothness constraints for $f_{B}$ are commonly applied to choose between multiple local minima.

#### 2.1.2 Compressed Sensing in WFS Reconstruction

Additional scan time introduced by the multi‐echo acquisition for WFS imaging can be compensated by integrating compressed sensing (CS) reconstruction applied to undersampled data [26]. CS leverages sparsity in a suitable transform domain and incoherent sampling to separate spatially aliased signal components. CS‐type regularization can be applied to reconstruct echo images as follows:

(3) $${\hat{x}}_{j}=\arg\min_{x_{j}}\;\|D_{j}\;F\;S\;x_{j}-y_{j}{\|}_{2}^{2}+\lambda \|T_{x}\;x_{j}{\|}_{1},$$

where $D_{j}$ is a sampling mask at echo j, F denotes the discrete Fourier transform (DFT), S is the coil sensitivity map estimated from the spin‐echo image, $x_{j}=x(t_{j})$ denotes the j‐th gradient echo image, $y_{j}$ denotes the multichannel k‐space signal, $T_{x}$ represents a Wavelet or other sparsifying transform applied to echo images, and λ is the regularization strength. Following CS reconstruction of the individual echo images, field mapping and species separation are applied according to Equation (1). This sequential workflow is therefore termed the “two‐stage” method.

An alternative to the two‐stage method is a “joint” reconstruction of multi‐echo data, where WFS images are extracted directly from undersampled k‐space using precomputed field‐ and $R_{2}^{\prime }$‐map estimates forming $\hat{\mu }$ [27].

(4) $$\begin{array}{ccc} \hat{\rho }=\arg\min_{\rho }\;\| & \underset{D}{\underbrace{\left(\begin{array}{c} D_{1}\\ \vdots \\ D_{N_{t}} \end{array}\right)}}\;F\;S\;A(\hat{\mu })\;\rho -\underset{y}{\underbrace{\left(\begin{array}{c} y_{1}\\ \vdots \\ y_{N_{t}} \end{array}\right)}}{\|}_{2}^{2}\;+\;\sum_{k=1}^{3}\lambda_{k}\;\|T_{k}\;\rho_{k}{\|}_{1}, & \end{array}$$

where $A(\hat{\mu })$ is the chemical‐shift encoding operator defined in Equation (1), k indexes the WFS species, and $T_{k}$ is a sparsifying transform applied directly to water, fat, or silicone images $\rho_{k}$.

The joint reconstruction offers two advantages over the two‐stage formulation. First, incorporating the chemical‐shift encoding matrix in the forward model enforces sparsity in the spectral (species) dimension and accounts for signal correlations across echoes. This effectively reduces the number of unknowns, allowing for more aggressive undersampling in the combined $k_{y}$–TE domain (Figures S1 and S3). Secondly, the joint method permits species‐specific CS regularizations, allowing the reconstruction to match the distinct spatial sparsity patterns of WFS (Figure S5). Furthermore, because the species signal magnitudes can differ substantially, the use of adaptive soft‐thresholding within $L_{1}$‐regularized reconstruction avoids over‐penalizing low‐intensity species. This improves structural preservation, particularly under high undersampling.

### 2.2 Acquisition

**FIGURE 1:**
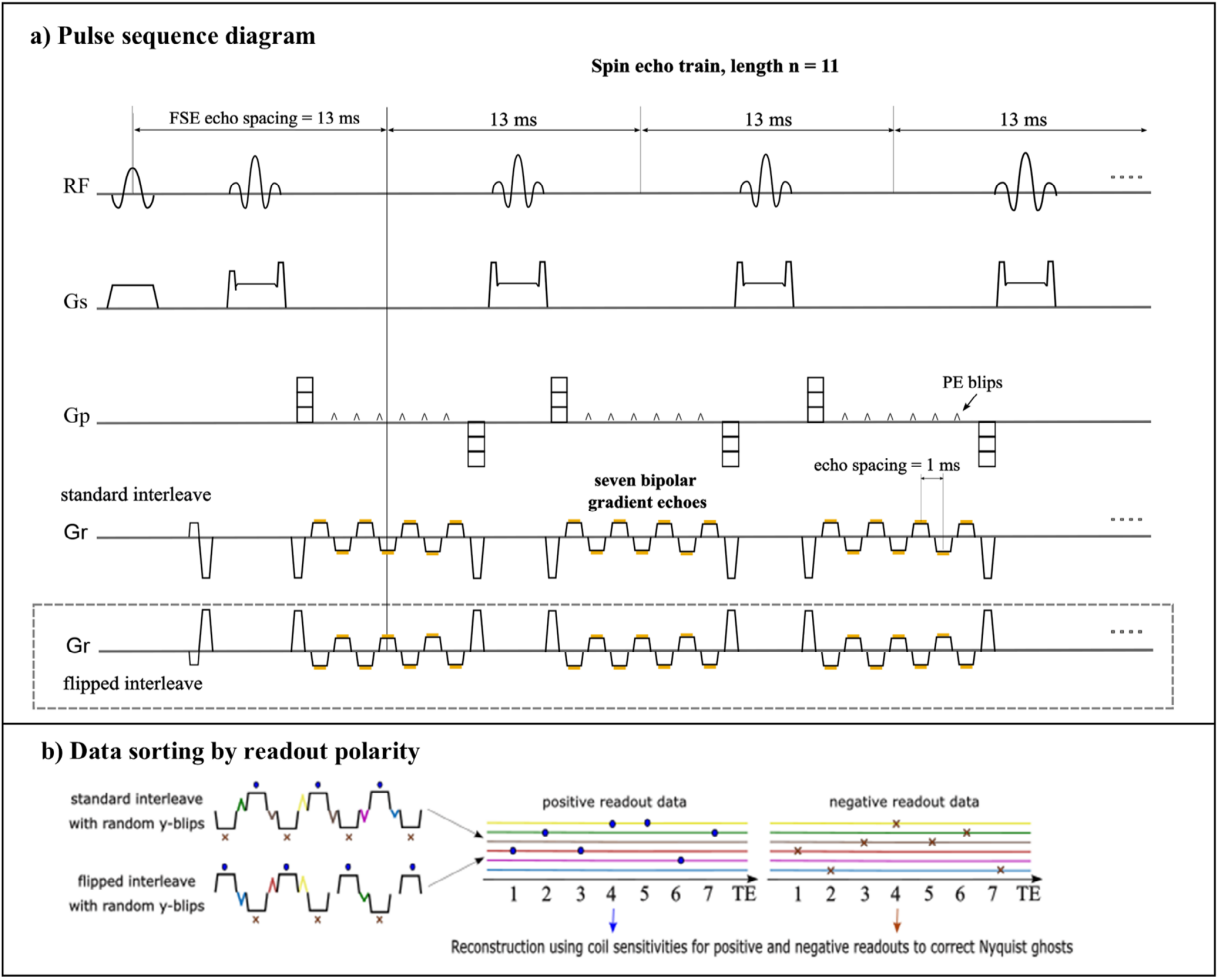
Proposed sequence diagram: (a) Multi‐echo 2D FSE sequence with incoherent undersampling in the $k_{y}$–TE domain. The spin‐echo train has an out‐in phase‐encoding ordering and 13 ms echo spacing. At each spin echo, seven bipolar gradient echoes are acquired with 1 ms spacing and small phase‐encoding blips in between to enable echo‐dependent $k_{y}$ sampling. A flipped interleaf with reversed readout polarity is also acquired. (b) Data from standard and flipped interleaves are grouped by polarity for coil sensitivity‐based Nyquist ghost correction and image reconstruction.

The proposed sequence was developed using the KS Foundation framework [28] with a 2D FSE template, incorporating seven bipolar readouts into each spin echo. The sequence design is illustrated in Figure 1a, and key parameters are summarized in Table 1. Readout crusher gradients were assigned a polarity opposite to that of the first echo to preserve eddy‐current phase consistency across the multi‐echo readout.

**TABLE 1:** Sequence parameters of the proposed $T_{2}$‐weighted multi‐echo 2D FSE and clinical reference STIR two‐echo Dixon 3D FSE method.

	Multi‐echo 2D FSE (proposed)	STIR two‐echo 3D FSE (reference)
TE [ms]	60	60
TR [s]	7	3
ETL, FSE ESP [ms]	11, 13	100, 3.5
Readouts per interleaf	seven‐echo	single‐echo
CSE ESP [ms]	1	1
PI	R=6	R=6
Resolution [mm]	1.0 × 1.5 × 2.0	1.5 × 1.5 × 2.0
Slice spacing [mm]	0, even‐odd slice interleaving	0, phase‐encoded
Number of slices	100	100
FOV, AP x LR x SI [mm]	360 × 360 × 200	240 × 360 × 200
Scan time [mins]	2.5	5
Reconstructed images: water (W), fat (F), silicone (S)	W, F, S, W+F, F+S, W+S, W+F+S	W, S, W+S

An additional interleaf with flipped readout polarities at each echo was acquired to correct for bipolar inconsistencies in the multi‐echo data. The interleaf beginning with positive‐readout polarity was defined as the **standard interleaf**, and the one beginning with negative polarity as the **flipped interleaf**. For central k‐space autocalibration signal (ACS) lines, both interleaves were acquired ($N_{\text{ACS}}=32$), while complementary undersampling was applied to the outer k‐space regions [29]. This design enabled low‐resolution field mapping (see “Data Processing”), and kept the calibration time minimal at ∼21seconds. The reported acceleration factors R are for the combined positive and negative data.

Incoherent $k_{y}$–TE undersampling pattern implementation followed [30], assigning odd/even temporal entries to standard/flipped interleaves, with $k_{y}$ constrained to change by ≤12Δk (where Δk is the $k_{y}$‐spacing) between adjacent echoes. On the sequence side, small y‐gradient blips were included between multi‐echo readouts, which resulted in spacing of 1.06ms using 50% ramp sampling.

The proposed method was compared to an established $T_{2}$‐weighted **clinical reference** method for imaging patients with silicone breast implants at our institution: the **STIR two‐echo Dixon 3D FSE**. This sequence included an optimized variable flip angle (VFA) [31] train and performed STIR [32] fat suppression with an inversion time of 220ms (for 3T). The two‐point Dixon was implemented in two interleaves with unipolar readouts, allowing separation of water and silicone signals in the absence of fat. Further sequence parameters are provided in Table 1.

### 2.3 Data Processing

**FIGURE 2:**
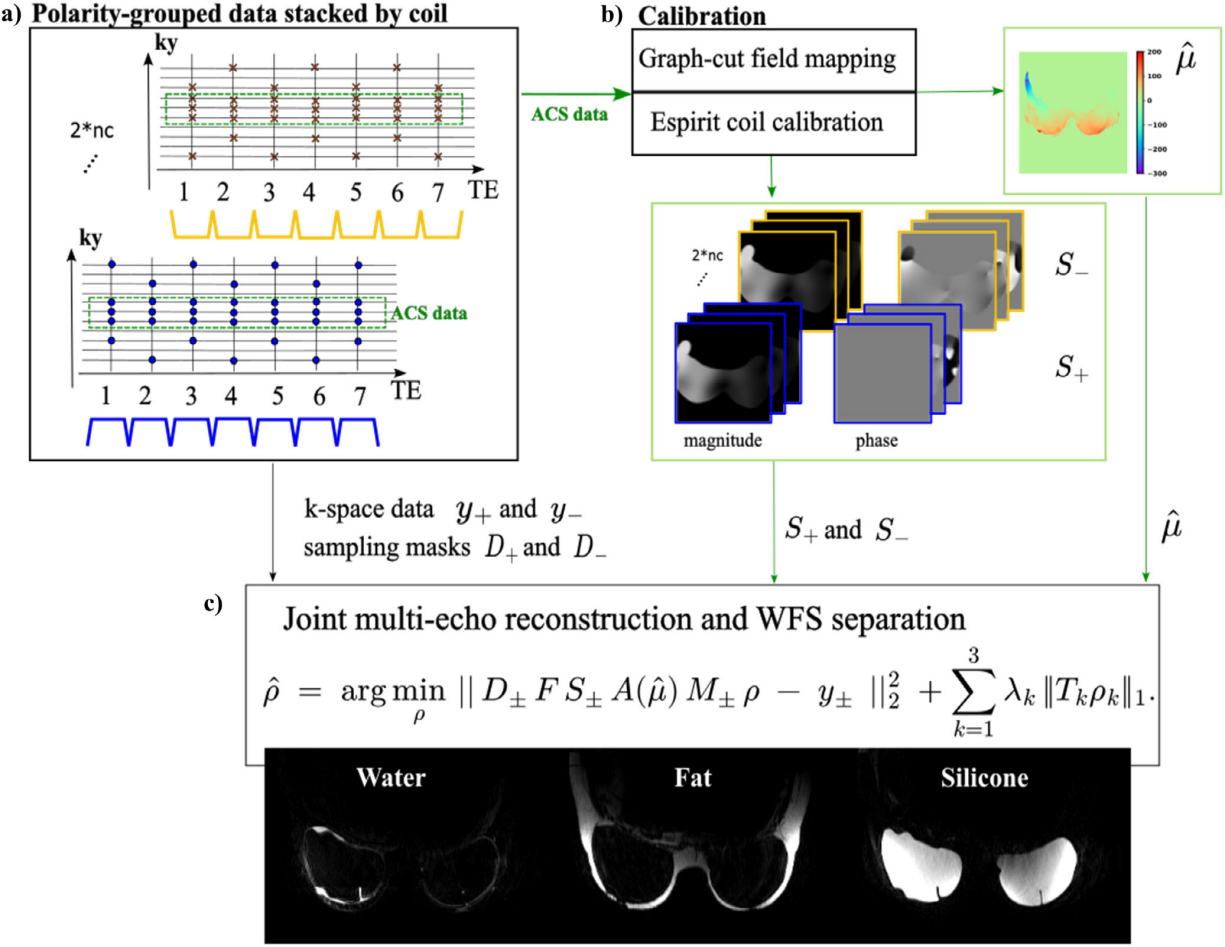
Proposed joint reconstruction framework for bipolar multi‐echo FSE data acquired with standard and flipped interleaves. (a) Readouts from the two interleaves are grouped by polarity and concatenated along the coil dimension. Since each polarity follows a distinct sampling pattern, the undersampling masks differ across coils. (b) A fully sampled ACS is used to derive coil sensitivity maps and low‐resolution field and $R_{2}^{\prime }$ maps forming $\hat{\mu }$. (c) The joint multi‐echo reconstruction and WFS separation directly estimates water, fat, and silicone images from the undersampled k‐space data. The forward model incorporates the pre‐estimated $\hat{\mu }$, the Nyquist ghost phase within $S_{\pm }$, the chemical‐shift blurring $M_{\pm }$, and the polarity‐specific sampling $D_{\pm }$ operators.

The pipeline is illustrated in Figure 2. First, the multi‐echo data were sorted to group k‐space matrices by readout polarities and stack the two polarity sets along the coil dimension (Figure 2a). Next, coil sensitivity maps for positive and negative polarities and a low‐resolution field map were computed from the ACS data (Figure 2b). Finally, the joint (Figure 2c) reconstruction was applied with Nyquist ghost and chemical‐shift blurring correction to obtain WFS images.

#### 2.3.1 Nyquist Ghost Correction (NGC)

Bipolar readouts were chosen for higher time efficiency, which was critical for achieving the required spectral encoding range in the WFS separation. However, eddy currents, gradient delays, and imperfect switching between positive (${\text{RO}}^{+}$) and negative (${\text{RO}}^{-}$) gradients introduced phase inconsistencies, resulting in Nyquist ghosting [33, 34]. We modeled these as spatially nonlinear phase differences [35] and estimated them within the ESPIRiT‐based coil calibration [36]. Specifically, ${\text{RO}}^{+}$ and ${\text{RO}}^{-}$ data were stacked along the coil dimension, enabling ESPIRiT to estimate coil phases relative to the first positive‐readout channel (Figure 2b). The resulting coil sensitivities were then used directly for SENSE‐like [37] coil and polarity combination:

(5) $$\begin{array}{c} {\hat{x}}_{\text{NGC}}=\arg\underset{x}{\min}\;\|\underset{D_{\pm }}{\underbrace{\left(\begin{array}{c} D_{+}\\ D_{-} \end{array}\right)}}\;F\;\underset{S_{\pm }}{\underbrace{\left(\begin{array}{c} S_{+}\\ S_{-} \end{array}\right)}}x-\underset{y_{\pm }}{\underbrace{\left(\begin{array}{c} y_{+}\\ y_{-} \end{array}\right)}}{\|}_{2}^{2}, \end{array}$$

where ${\hat{x}}_{\text{NGC}}$ represents the Nyquist ghost‐corrected images, and + and − subscripts denote positive/negative polarity readout.

#### 2.3.2 Chemical‐Shift Correction

The forward model in Equation (5) was further expanded to account for off‐resonant blurring due to opposing‐direction chemical shift in bipolar readouts. Because a field map is pre‐estimated in the joint reconstruction, the corresponding readout shifts of fat and silicone can be incorporated directly in the reconstruction. This is achieved by introducing image‐domain shift operators before the chemical‐encoding matrix $A(\hat{\mu })$. The operator $M_{\pm }$ stacks the positive and negative shift directions and has a block‐diagonal structure with respect to the species images ρ. Applying $A(\hat{\mu })$ after this operator acts identically on both components of the stacked representation:

(6) $$M_{\pm }=\left[\begin{array}{c} \mathrm{diag}\left(0,\;M(\Delta x_{f}),\;M(\Delta x_{s})\right)\\ \mathrm{diag}\left(0,\;M(-\Delta x_{f}),\;M(-\Delta x_{s})\right) \end{array}\right].$$

#### 2.3.3 Joint Reconstruction

Low‐resolution, Nyquist ghost–corrected multi‐echo images reconstructed from the calibration k‐space (${\hat{x}}_{\text{ACS}}$) were used to estimate the $\hat{\mu }$. Field mapping employed the open‐source hmrGC library [13], using a single layer of field mapping ($f_{B}$) and $R_{2}^{\prime }$‐mapping based on a silicone single‐peak model at −4.9ppm and the nine‐peak fat model from [18]. Species images were then reconstructed by solving the following problem:

(7) $$\begin{array}{ll} \hat{\rho }\; & =\arg\min_{\rho }\;\|\;D_{\pm }\;F\;S_{\pm }\;A(\hat{\mu })\;M_{\pm }\;\rho \;-\;y_{\pm }\;{\|}_{2}^{2}\;+\;\lambda_{w}\|\rho_{w}{\|}_{1}\\ & \;+\;\lambda_{f}\|W\rho_{f}{\|}_{1}\;+\;\lambda_{s}\|W\rho_{s}{\|}_{1}. \end{array}$$

The species‐specific CS regularization weights $\lambda_{w}$, $\lambda_{f}$, and $\lambda_{s}$ were selected for each dataset via a 3D hyperparameter grid search. Identity, Wavelet, and Wavelet transforms were chosen as sparsifying operators for the WFS images, respectively, based on compressibility analysis following Reference [26]. We observed that the water images are compressible in the image, TV, and Wavelet domains, and that fat and silicone images are compressible in the TV and Wavelet domains (Figure S5).

The baseline two‐stage reconstruction was implemented with $L_{1}$‐regularization on a Wavelet transform W and a single λ for the multi‐echo images. Similar to the joint method, we applied a single graph‐cut layer followed by $R_{2}^{\prime }$‐mapping, with similar fat and silicone spectral models.

### 2.4 Experiments

Fifteen subjects (10 with silicone implants, 1 with saline implants, 1 with saline+silicone implants, and 3 without implants) were imaged on a 3T GE Signa Premier system using a 16‐channel Sentinelle breast coil. All volunteers were scanned under IRB approval with informed consent. Next to the proposed $T_{2}$‐weighted multi‐echo 2D FSE acquisition, STIR two‐echo Dixon 3D FSE sequences were acquired with detailed scan parameters provided in Table 1. One more subject with silicone implants was imaged **supine** using a 60‐channel GE research breast coil [38]. For this case, breath‐holds were performed for our acquisition, and a respiratory‐triggered two‐point Dixon 3D FSE was used as the reference.

Retrospective and prospective undersampling experiments were performed in five subjects. Fully sampled datasets, including both standard and flipped interleaves, were acquired for reference. For quantitative evaluation, WFS images from joint reconstruction with uniform, variable‐density $k_{y}$‐only, and incoherent $k_{y}$–TE undersampling patterns were compared against the fully sampled reference using structural similarity index (SSIM) [39], peak signal‐to‐noise ratio (PSNR), and gradient magnitude similarity deviation (GMSD) [40]. The same metrics were applied to compare two‐stage versus joint reconstruction under incoherent $k_{y}$–TE undersampling. Additionally, we performed reconstruction benchmarks against previously published work [29] using uniformly undersampled R=6 bipolar data.

Further, we compared joint reconstruction with incoherent $k_{y}$–TE sampling at R=6 to the clinical reference qualitatively, and quantified water/fat and silicone signal “leakage”. Water/fat “leakage” was defined as the ratio of mean signal intensities in regions of interest (ROIs) outside versus inside the implant on the silicone image $I_{S}$:

(8) $$\frac{S_{WF}}{S}=\frac{\sum_{r\in {\text{ROI}}_{WF}}I_{S}(r)}{\sum_{r\in {\text{ROI}}_{S}}I_{S}(r)}\times 100\%.$$

Silicone signal “leakage” into water and fat images was defined as the ratio of mean signal in the implant ROI (${\text{ROI}}_{S}$) on the water $I_{W}$ and fat $I_{F}$ images relative to the silicone image $I_{S}$:

(9) $$\frac{W}{S}=\frac{\sum_{r\in {\text{ROI}}_{S}}I_{W}(r)}{\sum_{r\in {\text{ROI}}_{S}}I_{S}(r)}\times 100\%.$$

## 3 Results

### 3.1 Undersampling Experiments

Quantitative results from the retrospective undersampling experiment (Figure S3a) show that both variable‐density $k_{y}$ and incoherent $k_{y}$–TE sampling improve reconstruction quality across all species, most notably for water, yielding higher SSIM and PSNR, with median gains of 0.04–0.07 and 2–7 dB, and GMSD decreased by 0.03–0.06 compared to uniform $k_{y}$ sampling. Incoherent $k_{y}$–TE outperforms both uniform $k_{y}$ and variable‐density $k_{y}$ patterns for water and fat images, while improvements for silicone are less consistent.

Prospective undersampling experiments (Figure S4) confirm these findings. At acceleration R=6, incoherent $k_{y}$–TE sampling with joint reconstruction (d) produces the sharpest and most uniform WFS images, with minimal artifacts across species. In contrast, uniform $k_{y}$ undersampling (b) introduces coherent ghosting in water and silicone, while variable‐density $k_{y}$ sampling (c) causes blurring and background noise.

#### 3.1.1 Previous Works vs Proposed Approach

Benchmarking against Reference [29] with uniform R=6 undersampling (Figure S6) demonstrates that the proposed joint reconstruction provides comparable image quality and enables correction of blurring arising from opposite‐direction chemical shifts in bipolar data.

In retrospective experiments at R=6 with incoherent $k_{y}$–TE sampling (Figure S3b), the two‐stage reconstruction was outperformed by the joint method, which improved mean SSIM by 0.05–0.13, PSNR by 5–10 dB, and reduced GMSD by approximately 0.05–0.07.

**FIGURE 3:**
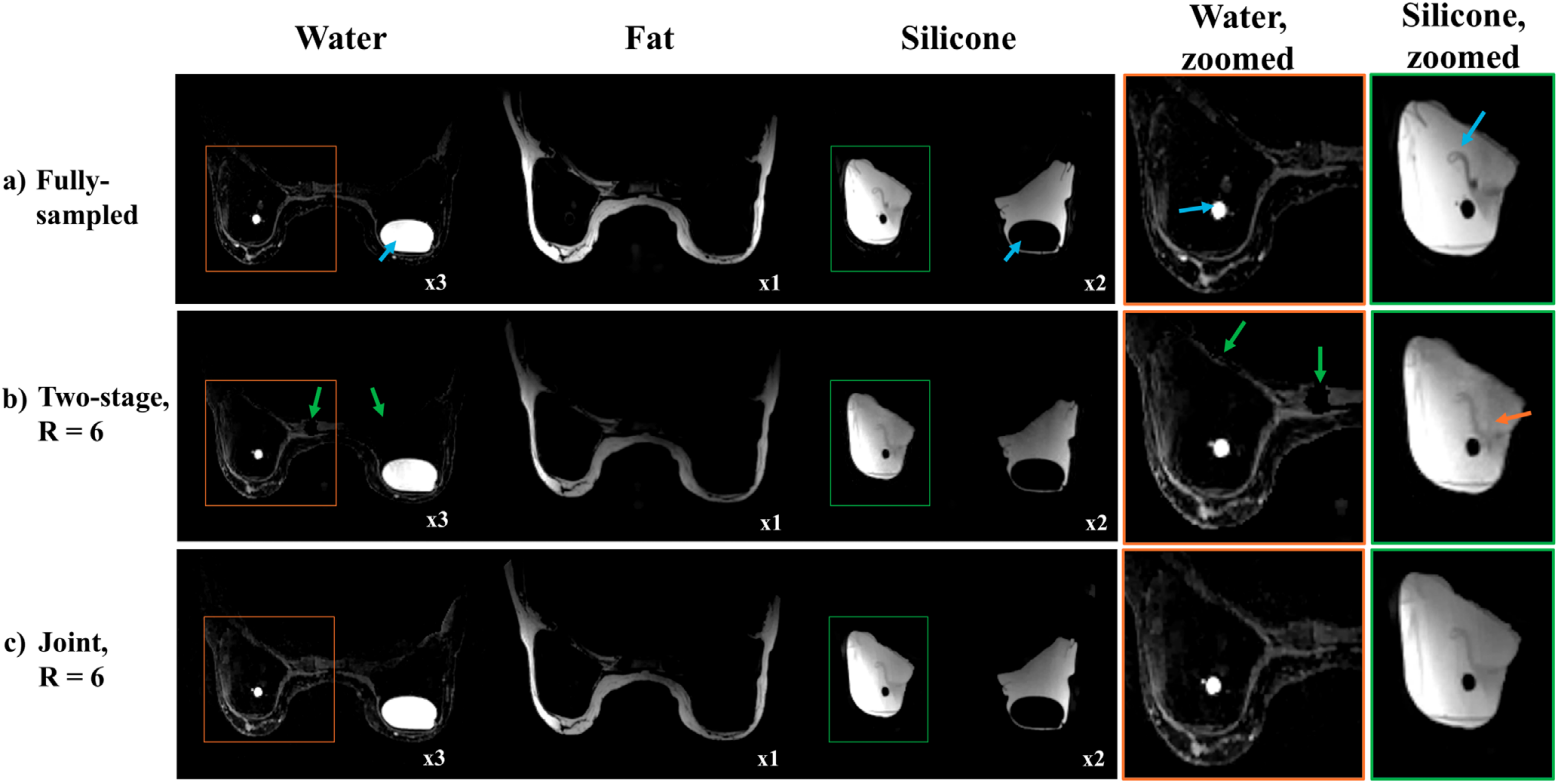
Comparison of two‐stage and joint reconstruction of multi‐echo data with incoherent $k_{y}$–TE undersampling at R=6 in a patient with a bilateral silicone implant rupture. Zoomed water and silicone views highlight the implant envelope collapse and internal water in both implants (blue arrows). (a) Fully sampled reference images. (b) Two‐stage reconstruction shows partial water signal loss (green arrows) and residual aliasing in silicone (orange arrows). (c) Joint reconstruction preserves structural details in water, provides improved regularization and artifact suppression in silicone images.

Figure 3 shows images from two‐stage and joint methods in a patient with bilateral implant rupture. Although both methods in (b)–(c) yield reasonable WFS images relative to the fully sampled reference in (a), the two‐stage approach shows structural loss in the water image near the chest wall (green arrows) and inferior denoising in silicone (orange arrow).

#### 3.1.2 Clinical Reference vs Proposed Approach

**FIGURE 4:**
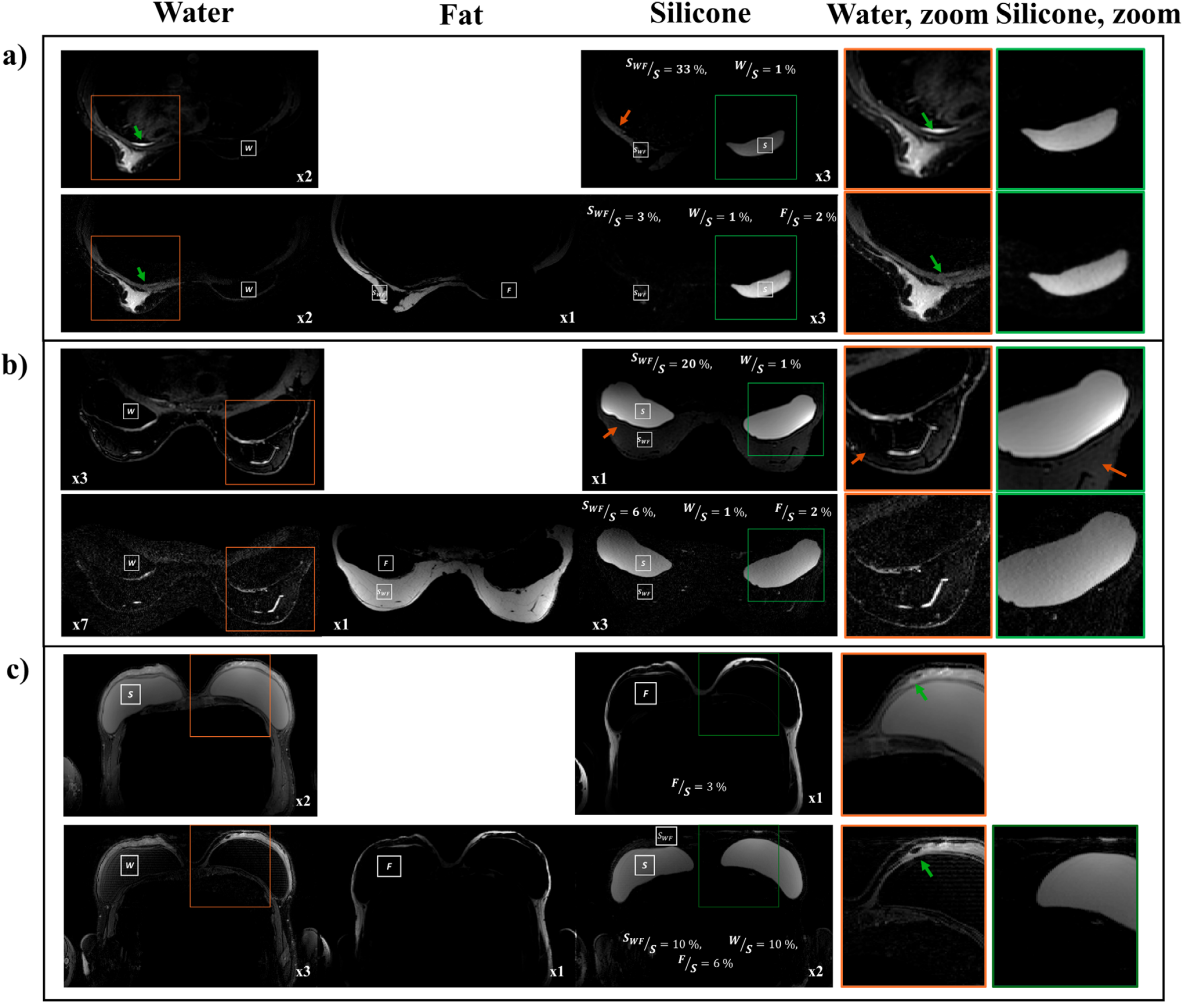
Comparison of water, fat, and silicone images from the clinical reference STIR two‐echo Dixon 3D FSE *(top rows)* and the proposed multi‐echo 2D FSE with incoherent $k_{y}$–TE sampling *(bottom rows)*. Shown are patients with (a) a unilateral silicone implant, (b) bilateral implants with fat‐flap overlay, and (c) bilateral implants. Cases (a–b) were acquired prone at R=6, while (c) was acquired supine under breath‐hold at R=4 (3.5 min) with our sequence, and with a respiratory‐triggered Dixon 3D FSE for the reference. The proposed method yields high‐resolution images with improved water–fat suppression ($\frac{S_{WF}}{S}=3\%$ and 6%) compared to the reference ($\frac{S_{WF}}{S}=20\%$ and 33%; red arrows). Zoomed‐in water and silicone images highlight the superior sharpness and lipid suppression achieved by the proposed approach.

Figure 4 summarizes three cases demonstrating the image quality and robustness of the proposed method. In case (a), the reference exhibits blurring and substantial water–fat leakage $\left(\frac{S_{WF}}{S}=33\%\right)$, whereas the proposed reconstruction reduces leakage to 3%. In case (b), with a fat‐flap overlying bilateral implants, the reference shows poor fat suppression, while the proposed method yields sharper water images and lowers water‐fat leakage from 20% to 6%.

Case (c), acquired supine with breath‐holds, maintains consistent species separation and sharp WFS images. The higher silicone‐to‐water and silicone‐to‐fat ratios $\left(\frac{W}{S}=10\%,\;\frac{F}{S}=6\%\right)$ relative to the prone scans may be due to motion.

**FIGURE 5:**
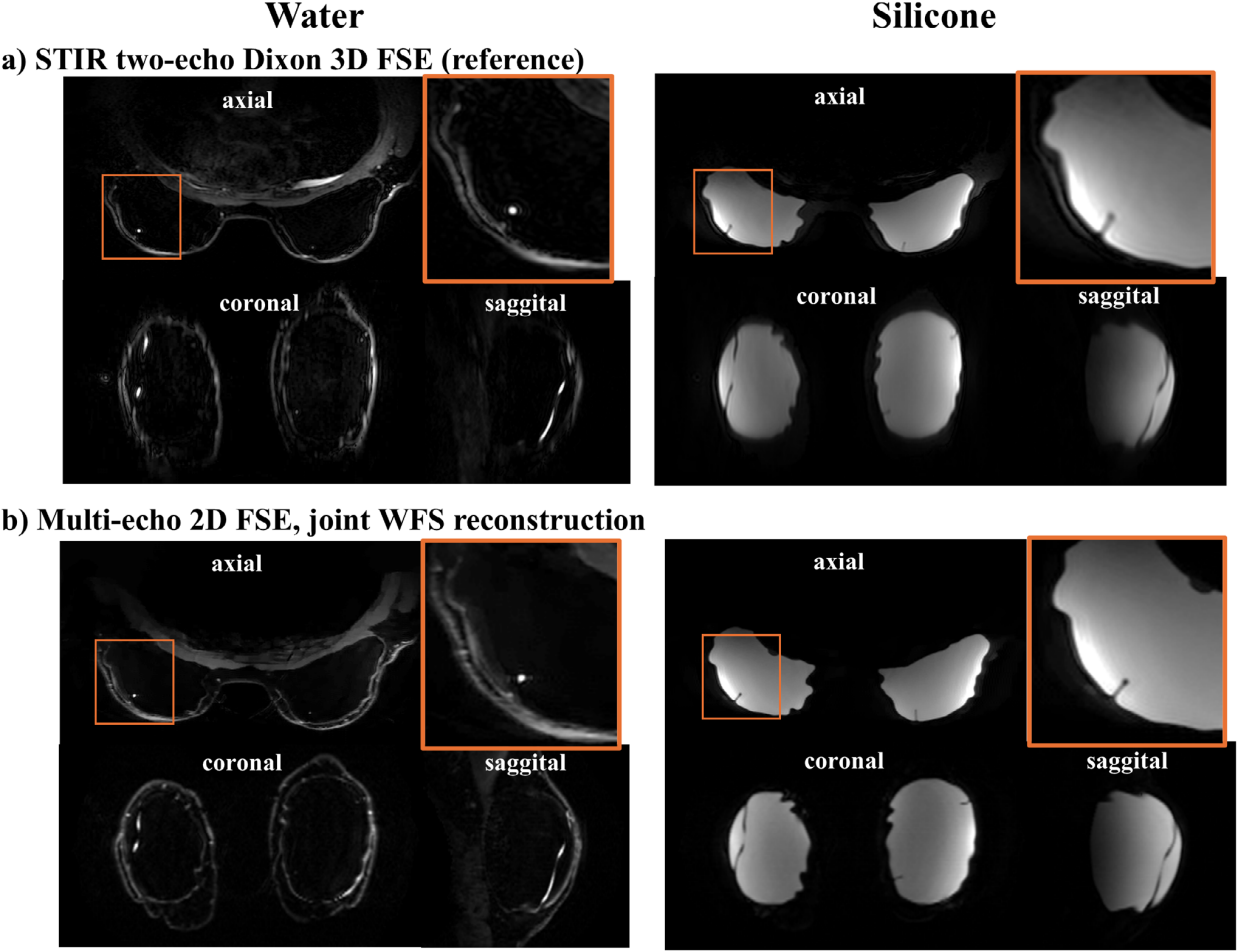
Comparison of three‐plane reformats for water and silicone images from (a) the reference STIR two‐echo 3D FSE and (b) the proposed multi‐echo 2D FSE with joint WFS reconstruction. The proposed method generates artifact‐free, co‐registered, and high‐resolution $T_{2}$‐weighted water, fat and silicone images in three planes.

Figure 5 compares water and silicone image reformats in three planes for the reference and proposed methods in a patient with bilateral silicone implants. Our method offers high‐resolution species images free of suppression failures and species swap artifacts. Water–fat suppression with our method is $\frac{S_{WF}}{S}=4\%$ compared to $\frac{S_{WF}}{S}=8\%$ with the reference.

## 4 Discussion

This study proposes a multi‐echo 2D FSE acquisition with echo‐dependent sampling and compressed sensing joint reconstruction for fast $T_{2}$‐weighted WFS imaging. The tightly spaced echoes and integrated NGC enabled reliable species separation and acceleration up to R=6. Compared with uniform and variable‐density $k_{y}$ sampling and with a conventional two‐stage method, the joint approach showed reduced ghosting and parallel‐imaging artifacts, and better preservation of low‐intensity water structures (Figures S1–S4). Relative to a STIR‐based 3D FSE reference, it produced comparable image quality in three planes and high resolution, while avoiding residual fat‐suppression artifacts and silicone displacement caused by frequency selective pulses.

The method resolves WFS images without suppression and also provides four combined “in‐phase” contrasts, improving boundary visualization and potentially eliminating the need for anatomical imaging sequences in conventional breast screening protocols for patients with implants (Figure S7). Performance is further consistent across subjects with and without implants (Figure S8).

Using bipolar readouts at each spin echo required NGC prior to WFS separation. We used the full ACS region ($N_{ky}=32$) to estimate the ${\text{RO}}^{+}/{\text{RO}}^{-}$ phase map, thus including nonlinear components in the correction [35]. Although joint estimation of ghost phase and image reconstruction is possible, we adopted a more stable pre‐estimation strategy requiring only grouping data by polarity. Intermediate NGC results are shown in Figure S6b,d.

With uniform $k_{y}$ undersampling, our acquisition follows the scheme of Reference [29]. In that work, reconstruction is performed in a two‐stage manner: multi‐echo images are first estimated separately for each bipolar polarity, followed by polarity combination and species separation. In this formulation, the Nyquist ghost phase appears as a field‐map offset. As suggested by comparisons with the two‐stage method, our formulation may support higher acceleration rates and additionally accounts for off‐resonant blurring from opposing‐direction chemical shift in bipolar readouts (Figure S6c–d).

The main limitation of our method is the 2D implementation, which resulted in lower SNR and greater motion sensitivity in comparison with the 3D reference. The joint method required empirical tuning of three regularization parameters; future work could explore automated selection strategies [41, 42, 43, 44] to make the reconstruction more practical. Finally, the joint reconstruction used low‐resolution field‐map estimates, which may be insufficient near strong $B_{0}$ variations, such as near metal, although this was not observed in our subjects.

## 5 Conclusions

This work presents a multi‐echo 2D FSE sequence with spatiotemporal undersampling and joint compressed sensing reconstruction for fast and robust $T_{2}$‐weighted imaging of WFS. The method enables higher accelerations and improved structural fidelity compared to conventional two‐stage reconstruction, allowing scan times of 2.5 minutes. Compared with a clinical reference STIR‐based two‐echo 3D FSE, the proposed method offers comparable or better resolution in all planes, robustness to field inhomogeneity, and avoidance of suppression‐related artifacts. With an explicit fat image and four “in‐phase” images for anatomical assessment, the presented approach enables robust, comprehensive diagnostic $T_{2}$‐weighted breast imaging regardless of implant presence.

## Funding

This work was supported by the Division of Cancer Prevention, National Cancer Institute (Grant No. R01‐CA249893), GE Healthcare, and the National Institute of Biomedical Imaging and Bioengineering (Grant No. R01‐EB009055).

## Conflicts of Interest

The authors declare no conflicts of interest.

## Supporting information




**Date S1: Table S1.** Comparison of silicone breast implant imaging methods. Notations: $\Delta B_{0}$—field inhomogeneities; **W, F, S**—water, fat, and silicone; high‐res—high resolution in three planes; SNR—signal‐to‐noise ratio.
**Figure S1.** PSF analysis for WFS reconstruction with different $k_{y}$–TE undersampling patterns. (a) Input signal and (b–f) PSFs for five joint‐reconstruction strategies: (b) uniform $k_{y}$ with constant TE, (c) uniform $k_{y}$ with diagonal TE increments, (d) variable‐density (VD) $k_{y}$ with constant TE, (e) VD $k_{y}$ with diagonal TE increments, (f) incoherent $k_{y}$–TE. Incoherent sampling (f) spreads aliasing across space and time, reducing ghosts compared with uniform (b–c) and VD (d–e) patterns with more structured aliasing.
**Figure S2.** Studied $k_{y}$–TE undersampling patterns. (a) With no phase‐encoding blips between echoes, grouping interleaves by readout polarity yields alternating $k_{y}$ patterns for odd vs. even echoes. (b) Positive‐readout patterns: uniform or variable‐density $k_{y}$ (both alternating in TE), and an incoherent $k_{y}$–TE pattern combining variable‐density $k_{y}$ with incoherent TE ordering.
**Figure S3.** Retrospective R=6 undersampling experiment: (a–c) comparison of three $k_{y}$–TE patterns for joint reconstruction; (d–f) two‐stage vs. joint reconstruction with incoherent sampling. SSIM, PSNR, and GMSD were computed against fully sampled WFS images. In (a–c), incoherent $k_{y}$–TE (green) yields the best overall metrics for water and fat, and improves SSIM/GMSD for silicone. In (d–f), joint reconstruction outperforms two‐stage for all species and metrics.
**Figure S4.** Prospectively undersampled reconstructions at R=6 from a patient with bilateral silicone implant rupture. (a) Fully sampled reference, (b) uniform $k_{y}$ sampling produces coherent ghosting in water and silicone (orange arrows), (c) variable‐density $k_{y}$ sampling introduces incoherent, noisy artifacts (orange arrows), (d) incoherent $k_{y}$–TE sampling reduces aliasing and most closely resembles the reference.
**Figure S5.** Wavelet‐domain compressibility of (a) water, (b) fat, and (c) silicone images. Uncompressed single‐species images (left) were compared to reconstructions using only the largest 20%, 10%, 5%, and 2.5% (left to right) of wavelet coefficients.
**Figure S6.** Coil‐based Nyquist Ghost Correction (NGC) results and comparison with [27] in a healthy volunteer with a silicone breast implant on a side. Water, fat, and silicone images, as well as difference maps between in‐phase images, are shown for: (a) unipolar data at R=2, (b) bipolar uncorrected data at R=3, (c) NGC using [27], (d) our proposed method. Uncorrected image in (b) shows parallel‐imaging and Nyquist ghost artifacts. Residual parallel‐imaging artifacts remain in (c), whereas our approach enables higher acceleration and integrates chemical‐shift correction directly within the reconstruction in (d).
**Figure S7.** Comparison of the proposed method with the current breast implant MRI protocol at our institution. The current protocol uses a $T_{1}$‐weighted 3D SPGR for anatomy/fat and a $T_{2}$‐weighted STIR 3D FSE for fluid‐sensitive tissue and implant assessment. The proposed multi‐echo 2D FSE with joint WFS reconstruction produces high‐resolution, co‐registered water‐, fat‐, and silicone‐only $T_{2}$‐weighted images plus four “in‐phase” combinations. This single scan provides a comprehensive evaluation of anatomy, pathology, and implant integrity while reducing scan time and improving robustness to $B_{0}$ variation.
**Figure S8.** Comparison of WFS images from the clinical reference and the proposed multi‐echo 2D FSE (R=4) in: (a–b) a patient with saline implant, (c–d) a patient with no implants. The proposed incoherent $k_{y}$–TE sampling with joint WFS reconstruction yields consistent, artifact‐free water–fat separation and an empty silicone estimate, while reducing scan time (3.5 vs. 5 min). Water images retain high‐resolution $T_{2}$‐weighted contrast, and fat images preserve co‐registered anatomy that is suppressed in the reference.
**Figure S9.** Robustness of the proposed joint water–fat–silicone reconstruction to varying shimming conditions. Separated WFS images and field maps are presented for dual‐ and single‐volume shimming prescriptions. The estimated field maps accounted for the changing linear shimming, resulting in nearly identical water, fat, and silicone separation.

## Data Availability

Reconstruction scripts will be publicly available under: https://github.com/nurdinova/water_fat_silicone.
