# Supplementary material for: T2‐Weighted Imaging of Water, Fat and Silicone
Source: Magn Reson Med. 2026 Jan 22;95(5):2765–75. doi: 10.1002/mrm.70253 (PMC12962225; doi:10.1002/mrm.70253)
Supplement: Supplementary file 1 — Date S1: Table S1. Comparison of silicone breast implant imaging methods. Notations: ΔB0—field inhomogeneities; W, F, S—water, fat, and silicone; high‐res—high resolution in three planes; SNR—signal‐to‐noise ratio. Figure S1. PSF analysis for WFS reconstruction with different ky–TE undersampling patterns. (a) Input signal and (b–f) PSFs for five joint‐reconstruction strategies: (b) uniform ky with constant TE, (c) uniform ky with diagonal TE increments, (d) variable‐density (VD) ky with constant TE, (e) VD ky with diagonal TE increments, (f) incoherent ky–TE. Incoherent sampling (f) spreads aliasing across space and time, reducing ghosts compared with uniform (b–c) and VD (d–e) patterns with more structured aliasing. Figure S2. Studied ky–TE undersampling patterns. (a) With no phase‐encoding blips between echoes, grouping interleaves by readout polarity yields alternating ky patterns for odd vs. even echoes. (b) Positive‐readout patterns: uniform or variable‐density ky (both alternating in TE), and an incoherent ky–TE pattern combining variable‐density ky with incoherent TE ordering. Figure S3. Retrospective R=6 undersampling experiment: (a–c) comparison of three ky–TE patterns for joint reconstruction; (d–f) two‐stage vs. joint reconstruction with incoherent sampling. SSIM, PSNR, and GMSD were computed against fully sampled WFS images. In (a–c), incoherent ky–TE (green) yields the best overall metrics for water and fat, and improves SSIM/GMSD for silicone. In (d–f), joint reconstruction outperforms two‐stage for all species and metrics. Figure S4. Prospectively undersampled reconstructions at R=6 from a patient with bilateral silicone implant rupture. (a) Fully sampled reference, (b) uniform ky sampling produces coherent ghosting in water and silicone (orange arrows), (c) variable‐density ky sampling introduces incoherent, noisy artifacts (orange arrows), (d) incoherent ky–TE sampling reduces aliasing and most closely resembles the reference. Figure S5. Wavelet‐dom [file MRM-95-2765-s001.pdf]

T_2-weighted Imaging of Water, Fat and Silicone

Supplementary Materials

S1 Choosing Undersampling Pattern

S1.1 PSF analysis

To define optimal k_y–TE undersampling for the joint multi-echo reconstruction, we analyzed aliasing and inter-species leakage with five spatio-temporal sampling patterns: two spatially uniform and three variable-density (VD) sampling schemes. Figure S1 illustrates the reconstruction point-spread function (PSF) for a delta-function input in the water channel at an acceleration factor of $R = 4$, defined by:

$$\hat{\rho} = A^H \, F^H \, \frac{1}{\Theta} \, (D \, F) \, A \, \delta(y_c, \text{water}) \tag{S1}$$

where y_c is the central coordinate along the phase-encoding dimension, and Θ is the density-compensation function (DCF).

With each sampling pattern, we calculated a maximum aliasing metric as the maximum difference between the PSF and the input signal, and a leakage ratio defined as the maximum signal appearing in the fat and silicone channels when the input contained water only:

$$\begin{aligned} \text{max\_aliasing} &= \max\{\hat{\rho}_w - \delta(y_c, \text{water})\}; \\ \text{leakage\_ratio} &= \max(\hat{\rho}_{f,s}). \end{aligned} \tag{S2}$$

With uniform sampling in (a), we observed the expected equispaced aliases within the same species, along with leakage to other species due to imperfect conditioning of the CSE system. In contrast, variable-density k_y sampling in (d) generated incoherent aliasing in the same species and scaled leakage into other species. When using incoherent k_y–TE sampling in (f), aliasing was distributed in the spatial and temporal dimensions, significantly reducing per-species aliasing. However, a particular challenge in WFS imaging is the substantially different signal magnitudes across the three species, which may result in fat leakage comparable to the water signal level itself. Uniform and variable-density diagonal sampling in time ((c) and (e)) exhibited intermediate aliasing and leakage, since these patterns are neither constant nor entirely randomized across echo times.

S1.2 Retrospective Undersampling Reconstruction

Three k_y–TE patterns were evaluated in undersampling experiments (Figure S2(b)). **Uniform $\mathbf{k_y}$** acquired $2R$-fold undersampled k-space for all gradient echoes in the standard interleaf. The flipped interleaf used the same pattern but was shifted by

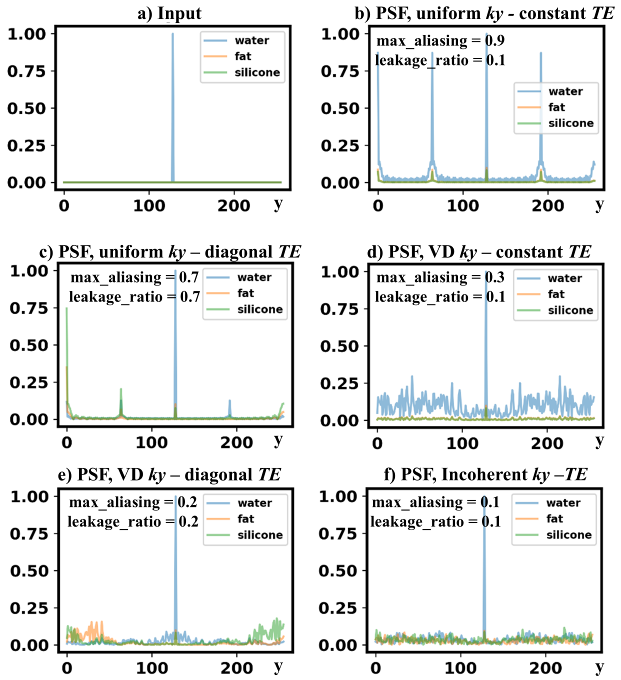

Figure S1: PSF analysis for WFS reconstruction with different k_y–TE undersampling patterns. (a) Input signal and (b–f) PSFs for five joint-reconstruction strategies: (b) uniform k_y with constant TE, (c) uniform k_y with diagonal TE increments, (d) variable-density (VD) k_y with constant TE, (e) VD k_y with diagonal TE increments, (f) incoherent k_y–TE. Incoherent sampling (f) spreads aliasing across space and time, reducing ghosts compared with uniform (b–c) and VD (d–e) patterns with more structured aliasing.

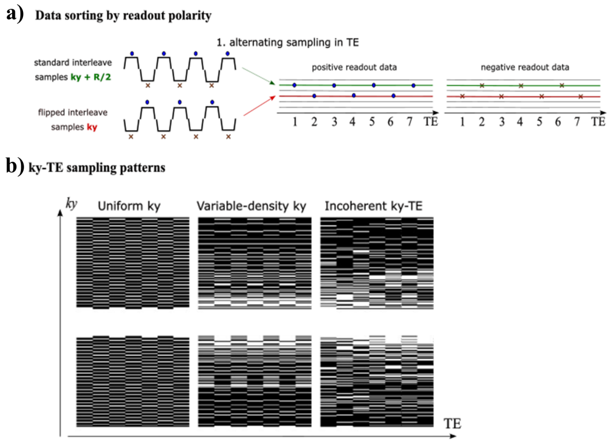

Figure S2: Studied k_y–TE undersampling patterns. (a) With no phase-encoding blips between echoes, grouping interleaves by readout polarity yields alternating k_y patterns for odd vs. even echoes. (b) Positive-readout patterns: uniform or variable-density k_y (both alternating in TE), and an incoherent k_y–TE pattern combining variable-density k_y with incoherent TE ordering.

R k_y lines to cover complementary lines in the outer k-space. Combining interleaves resulted in an effective acceleration factor of R, while leveraging Nyquist ghost correction using the ACS lines. Grouping positive and negative polarity readouts resulted in reconstruction data where odd and even echo times sampled complementary k-space lines in the outer k-space (Figure S2(a)). **Variable-density $\mathbf{k_y}$** sampling employed a Poisson sampling distribution along the y-axis, which remained consistent across all echo times. Standard and flipped interleaves sampled different k-space lines drawn from the same distribution. Therefore, after sorting the positive and negative readout data, the k-space sampling patterns differed slightly at odd and even echoes. **Incoherent $\mathbf{k_y}$–TE** sampling followed the method of [1], applied to merged echo–interleaf time points. Odd entries in the complementary-across-time mask were assigned to the standard interleaf; even entries to the flipped interleaf. The k_y values within each interleaf were constrained to vary by no more than $12 \times \Delta k$ between adjacent echoes in the multi-echo readout.

Undersampling experiments were performed in four subjects with silicone implants and one with saline implants. For this purpose, datasets with fully sampled (including both standard and flipped interleaves) and $R = 6$ undersampled acquisitions were obtained using uniform k_y, VD k_y, and incoherent k_y–TE sampling patterns.

Fully sampled data were retrospectively undersampled and reconstructed for quantitative evaluation of the three k_y–TE sampling strategies with two-stage and joint methods. Resulting water–fat–silicone (WFS) images were compared to the fully sampled reference using the structural similarity index (SSIM) [2], peak signal-to-noise ratio (PSNR), and gradient magnitude similarity deviation (GMSD) [3] metrics. Prospectively undersampled datasets were reconstructed using joint and two-stage methods and evaluated qualitatively, as motion between acquisitions precluded voxel-wise comparison across sampling patterns.

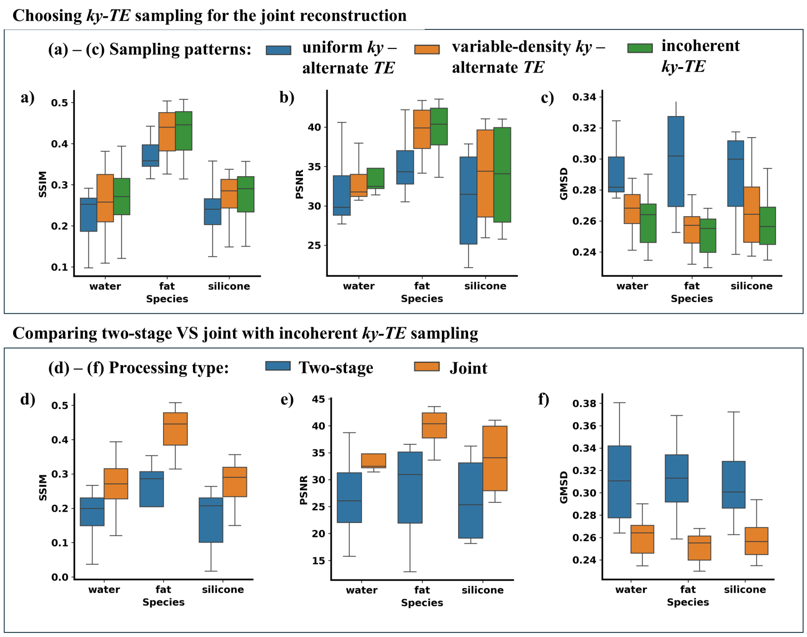

Figure S3: Retrospective $R = 6$ undersampling experiment: (a–c) comparison of three k_y–TE patterns for joint reconstruction; (d–f) two-stage vs. joint reconstruction with incoherent sampling. SSIM, PSNR, and GMSD were computed against fully sampled WFS images. In (a–c), incoherent k_y–TE (green) yields the best overall metrics for water and fat, and improves SSIM/GMSD for silicone. In (d–f), joint reconstruction outperforms two-stage for all species and metrics.

Quantitative results from the retrospective undersampling experiment (Figure S3(a)) show that both variable-density k_y and incoherent k_y–TE sampling improve reconstruction quality across all species—most notably for water—yielding higher SSIM and PSNR, and lower GMSD compared to uniform k_y sampling. Incoherent k_y–TE outperforms both alternating-in-TE uniform k_y and VD k_y patterns for water and fat images, while improvements for silicone are less consistent.

Figure S3(b) shows that at $R = 6$, the joint method with incoherent k_y–TE undersampling yields visibly superior reconstruction in terms of SSIM, PSNR, and GMSD metrics compared to the two-stage pipeline across the three species.

Prospective undersampling experiments (Figure S4) confirm these findings. Incoherent k_y–TE sampling with joint reconstruction (d) produces the sharpest and most uniform WFS images, with minimal artifacts across species. In contrast, uniform k_y undersampling (b) introduces coherent ghosting in water and silicone, while variable-density k_y sampling (c) causes blurring and background noise.

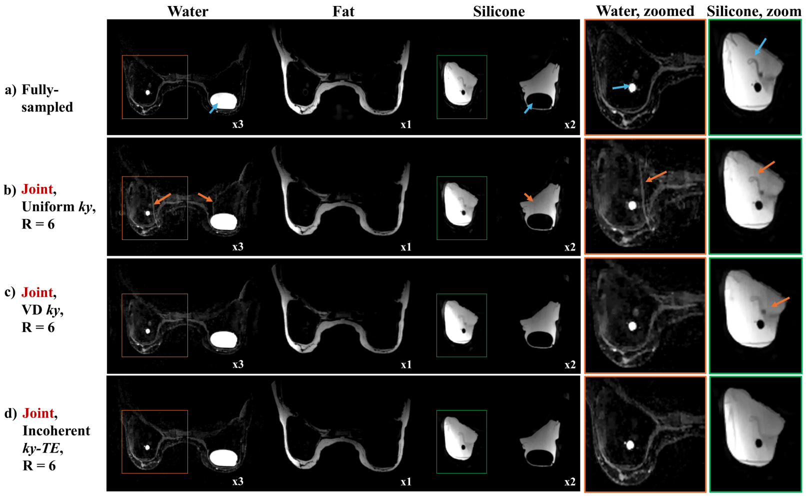

Figure S4: Prospectively undersampled reconstructions at $R = 6$ from a patient with bilateral silicone implant rupture. (a) Fully sampled reference, (b) uniform k_y sampling produces coherent ghosting in water and silicone (orange arrows), (c) variable-density k_y sampling introduces incoherent, noisy artifacts (orange arrows), (d) incoherent k_y–TE sampling reduces aliasing and most closely resembles the reference.

S1.3 Compressibility of WFS Images

Wavelet-domain analysis in Figure S5 demonstrates varying compressibility across WFS species. Water and fat images retained visually accurate detail with only 10% of wavelet coefficients, while silicone images remained stable even at 5%. Structured residuals became more prominent at higher compression levels (5% and 2.5%), particularly affecting anatomical detail and edge sharpness. Green boxes indicate the lowest coefficient ratios that preserved acceptable image quality for each species.

S2 Nyquist Ghost Correction

We demonstrate the effectiveness of NGC and compare our approach with [4] in Figure S6, using data from a healthy volunteer with a silicone implant adjacent to the right breast. Water, fat, and silicone images from (a) unipolar data at acceleration $R = 2$ are compared with those from (b) interleaved bipolar acquisition at $R = 3$. Difference maps (shown at $4\times$ intensity) highlight FOV/6 Nyquist ghosts without correction, which are substantially reduced after applying NGC in (d). Residual edge patterns in (b) and (c) arise from off-resonant blurring inherent to bipolar data combination. Compared with [4], our method shows fewer parallel imaging artifacts and effectively corrects for bipolar chemical shift effects, as demonstrated in Figure S6(c–d).

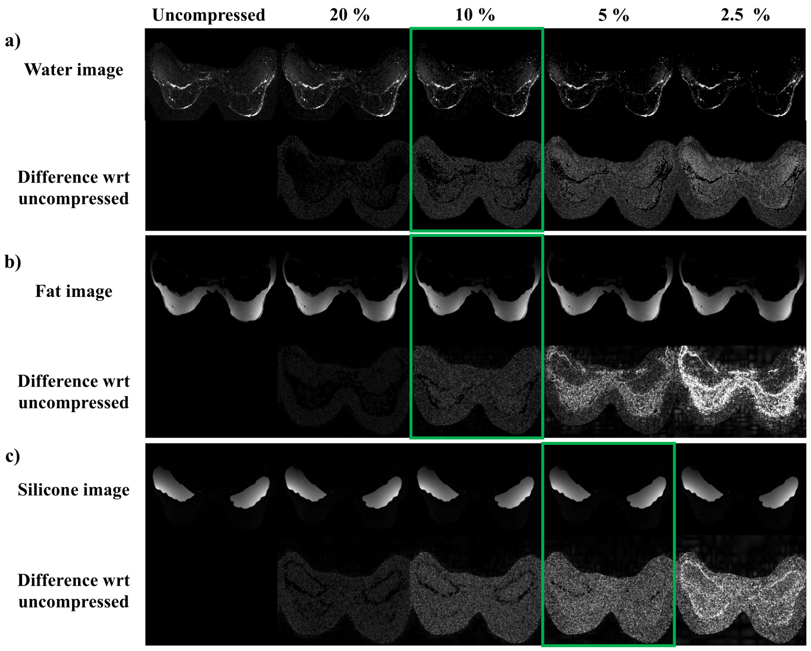

Figure S5: Wavelet-domain compressibility of (a) water, (b) fat, and (c) silicone images. Uncompressed single-species images (left) were compared to reconstructions using only the largest 20%, 10%, 5%, and 2.5% (left to right) of wavelet coefficients.

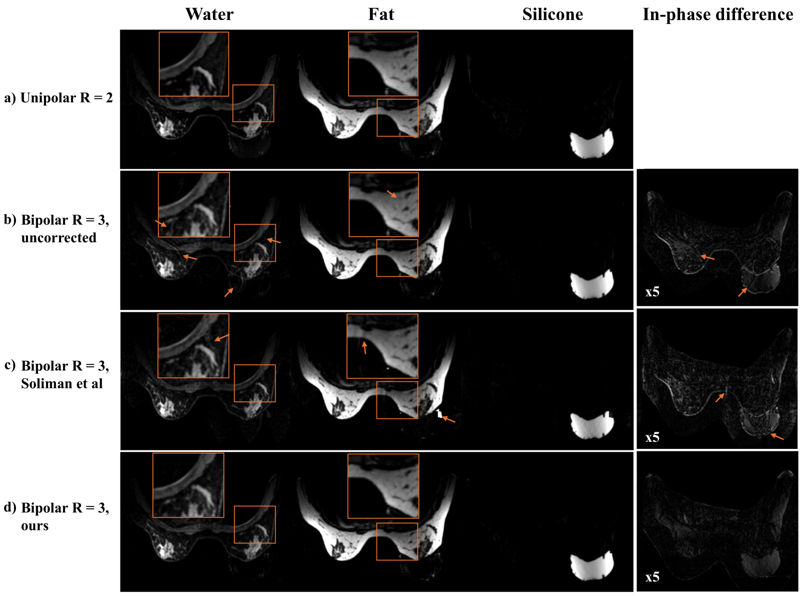

Figure S6: Coil-based Nyquist Ghost Correction (NGC) results and comparison with [4] in a healthy volunteer with a silicone breast implant on a side. Water, fat, and silicone images, as well as difference maps between in-phase images, are shown for: (a) unipolar data at $R = 2$, (b) bipolar uncorrected data at $R = 3$, (c) NGC using [4], and (d) our proposed method. The uncorrected image in (b) shows parallel imaging and Nyquist ghost artifacts. Residual parallel imaging artifacts remain in (c), whereas our approach enables higher acceleration and integrates chemical-shift correction directly within the reconstruction in (d).

S3 Simplifying MR Protocols

To illustrate how the proposed method streamlines breast MRI screening protocols, we present results in (1) subjects with saline implants and without implants, (2) under different shimming conditions, and (3) a side-by-side comparison to existing acquisitions.

Uniform performance is demonstrated in Figure S8 with example images from a saline-implant case and one without implants. Since no silicone is present, only water and fat images from the reference STIR two-echo Dixon 3D FSE are compared with those from the proposed multi-echo 2D FSE using joint reconstruction. In contrast to reference methods that suppress fat when a silicone-specific acquisition is used, our reconstruction produces water, fat, and an empty silicone channel in subjects without silicone, preserving consistent output across patients. Water images provide high-resolution T_2-weighted contrast, while fat images highlight co-registered anatomy.

To assess robustness to shim variability, data were acquired in a healthy volunteer with a silicone implant placed adjacent to the left breast under two shimming conditions (Figure S9). Case (a) used an optimal dual-volume shim covering both the breast and implant, whereas case (b) employed a broad single-volume shim extend-

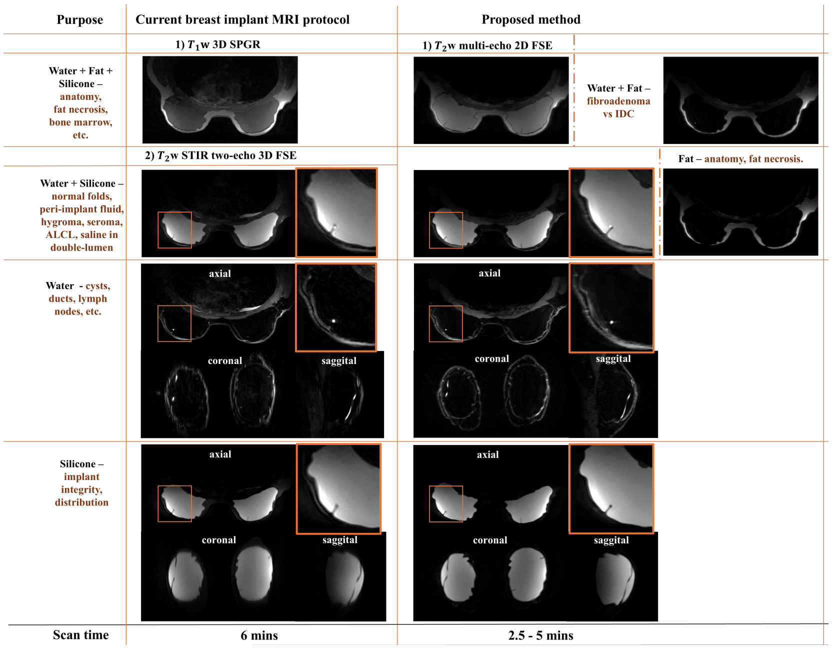

Figure S7: Comparison of the proposed method with the current breast implant MRI protocol at our institution. The current protocol uses a T_1-weighted 3D SPGR for anatomy/fat and a T_2-weighted STIR 3D FSE for fluid-sensitive tissue and implant assessment. The proposed multi-echo 2D FSE with joint WFS reconstruction produces high-resolution, co-registered water-, fat-, and silicone-only T_2-weighted images plus four "in-phase" combinations. This single scan can provide comprehensive evaluation of anatomy, pathology, and implant integrity while reducing scan time and improving robustness to B_0 variation.

Method	Advantages	Disadvantages
STIR two-point Dixon 3D FSE[5]	T_2-weighted, 3D, high-res, **W** and **S** images, robust to ΔB_0	no **F** image, limited SNR, residual fat, scan time 5–9 mins
TIMGRE[6]	3D, high-res, **W, F, S** images, robust to ΔB_0, fast (4 mins)	**not** T_2-weighted
Water-suppressed STIR 2D FSE[7]	T_2-weighted, **S** image	2D, low slice-res, no **W** and **F** image, sensitive to ΔB_0, limited SNR, long scan time
DIR 2D FSE[8, 9]	T_2-weighted, **S** image	
3-point Dixon 2D FSE[10]	T_2-weighted, **S**, **W+F** images, robust to ΔB_0	2D, low slice-res, shown at 1.5T only
Proposed	T_2-weighted, high-res, **W, F, S** images, robust to ΔB_0, fast (2.5–5 mins)	2D

Table S1: Comparison of silicone breast implant imaging methods. Notations: ΔB_0 – field inhomogeneities, **W, F, S** – water, fat, and silicone; high-res – high resolution in three planes; SNR – signal-to-noise ratio.

ing into the lung. Linear shim values recorded at the scanner showed changes of 4 and -8 Hz/cm between the two settings along the x- and z-directions, respectively, resulting in approximately 80 and -160 Hz variation across the prescribed FOV.

Despite these differences, the field-map estimation compensated effectively for the shim variability, yielding nearly identical separated water, fat, and silicone images. Silicone leakage into water and fat was quantified at $\sim 1\%$ and $\sim 3\%$, respectively, while water–fat leakage into silicone remained $\sim 2\%$ in both cases.

The proposed T_2-weighted acquisition simultaneously resolves water, fat, and silicone without suppression, enabling comprehensive evaluation in patients with implants. As shown in Figure S7, the 2.5–5 minute scan produces water-, fat-, and silicone-specific images, along with four combined "in-phase" contrasts. These include a full in-phase (W+F+S) image for overall anatomy and a water+silicone composite useful for differentiating implant rupture from benign peri-implant fluid. Additional fat and water+fat contrasts, unique to our method and free from fat–silicone swaps, enhance visualization of tissue boundaries and may aid in assessing lesions such as fibroadenomas, invasive ductal carcinoma, or bone marrow infiltration. Thus, a single, short acquisition achieves diagnostic coverage comparable to one or two conventional sequences while remaining robust to B_0 inhomogeneity and implant presence. All

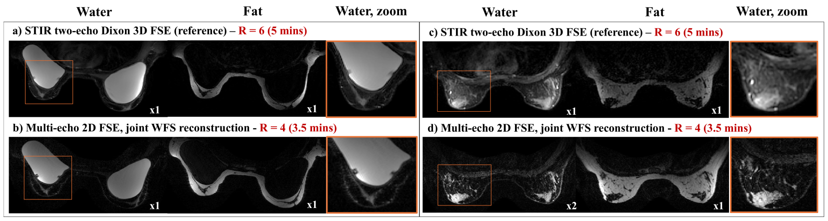

Figure S8: Comparison of WFS images from the clinical reference and the proposed multi-echo 2D FSE ($R = 4$) in: (a–b) a patient with saline implants; (c–d) a patient with no implants. The proposed incoherent k_y–TE sampling with joint WFS reconstruction yields consistent, artifact-free water–fat separation and an empty silicone estimate, while reducing scan time (3.5 vs. 5 min). Water images retain high-resolution T_2-weighted contrast, and fat images preserve co-registered anatomy that is suppressed in the reference.

images are high-resolution in three planes (water and silicone reformats shown in Figure S7) and free of suppression failures or species-swap artifacts.

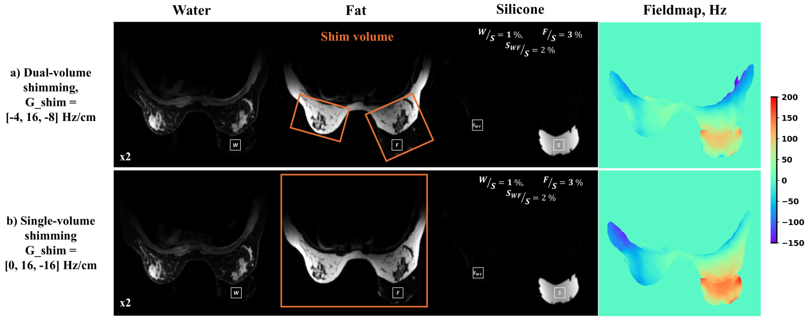

Figure S9: Robustness of the proposed joint water–fat–silicone reconstruction to varying shimming conditions. Separated WFS images, and field maps are presented for dual- and single-volume shimming prescriptions. The estimated field maps accounted for the changing linear shimming, resulting in nearly identical water, fat, and silicone separation results.

References

[1] Peng Lai, Anja Brau. Improving cardiac cine MRI on 3T using 2D k-t accelerated auto-calibrating parallel imaging. *Journal of Cardiovascular Magnetic Resonance* 2014. 16(Suppl 1):W3.

[2] Zhou Wang, Bovik, A.C., Sheikh, H.R., Simoncelli, E.P.. Image quality assessment: from error visibility to structural similarity. *IEEE Transactions on Image Processing* 2004. 13(4):600-612.

[3] Xue, Wufeng, Zhang, Lei, Mou, Xuanqin, Bovik, Alan C.. Gradient Magnitude Similarity Deviation: A Highly Efficient Perceptual Image Quality Index. *IEEE Transactions on Image Processing* 2014. 23(2):684-695.

[4] Soliman, Ahmed S., Wiens, Christopher N., Wade, Trevor P., McKenzie, Charles A.. Fat quantification using an interleaved bipolar acquisition. *Magnetic Resonance in Medicine* 2016. 75(5):2000–2008. doi:10.1002/mrm.25807.

[5] Jingfei Ma, Haesun Choi, R. Jason Stafford, Michael J. Miller. Silicone-Specific Imaging Using an Inversion-Recovery-Prepared Fast Three-Point Dixon Technique. *Journal of Magnetic Resonance Imaging* 2004. 19(3):298-302.

[6] Jonathan K. Stelter, Christof Boehm, Stefan Ruschke, Kilian Weiss, Maximilian N. Diefenbach, Mingming Wu, Tabea Borde, Georg P. Schmidt, Marcus R. Makowski, Eva M. Fallenberg, Dimitrios C. Karampinos. Hierarchical Multi-Resolution Graph-Cuts for water, fat and silicone Separation in Breast MRI. *IEEE Transactions on Medical Imaging* 2022. 41(11):3253-3265.

[7] Silva, Anabela, Oliveira, Mónica, Rocha, Humberto, Martinho, Miguel, Ferreira, Hugo A.. Developing a Specific MRI Silicone Imaging Sequence Using Inversion Recovery Techniques. *Applied Sciences* 2021. 11(8):3434.

[8] Mukundan Jr, S., Dixon, W. T., Kruse, B. D., Monticciolo, D. L., Nelson, R. C.. MR imaging of silicone gel-filled breast implants in vivo with a method that visualizes silicone selectively. *Journal of Magnetic Resonance Imaging* 1993. 3(5):713–717.

[9] Monticciolo, D. L., Nelson, R. C., Dixon, W. T., Bostwick, J., Mukundan, S., Hester, T. R.. MR detection of leakage from silicone breast implants: Value of a silicone-selective pulse sequence. *American Journal of Roentgenology* 1994. 163(1):51–56.

[10] Schneider, Erika, Chan, Teresa W.. Selective MR Imaging of Silicone with the Three-point Dixon Technique. *Radiology* 1993. 187(1):89–93.

[11] Plastic Surgery Statistics Report 2020. 2020.

[12] Brian D. Norena-Rengifo, Maria Paulina Sanin-Ramirez, Beatriz E. Adrada, Ana Beatriz Luengas, Vicente Martinez de Vega, Mary S. Guirguis, Cristina Saldarriaga-Uribe. MRI for Evaluation of Complications of Breast Augmentation. *Radiographics* 2022. 42(4):929-946.

[13] Reed F. Busse, Hari Hariharan, Anthony Vu, Jean H. Brittain. Fast spin echo sequences with very long echo trains: Design of variable refocusing flip angle schedules and generation of clinical T2 contrast. *Magnetic Resonance in Medicine* 2006. 55(5):1030-1037.

[14] Scott B Reeder, Angel R Pineda, Zhifei Wen, Ann Shimakawa, Huanzhou Yu, Jean H Brittain, Garry E Gold, Christopher H Beaulieu, Norbert J Pelc. Iterative decomposition of water and fat with echo asymmetry and least-squares estimation (IDEAL): Application with fast spin-echo imaging. *Magnetic Resonance in Medicine* 2005. 54(3):636-644.

[15] Jessica A. McKay, Steen Moeller, Lei Zhang, Edward J. Auerbach, Michael T. Nelson, Patrick J. Bolan. Nyquist ghost correction of breast diffusion weighted imaging using referenceless methods. *Magnetic Resonance in Medicine* 2019. 81(4):2624-2631.

[16] Martin Uecker, Peng Lai, Mark J. Murphy, Patrick Virtue, Michael Elad, John M. Pauly, Shreyas S. Vasanawala, Michael Lustig. ESPIRiT - An eigenvalue approach to autocalibrating parallel MRI: Where SENSE meets GRAPPA. *Magnetic Resonance in Medicine* 2014. 71(3):990-1001.

[17] Klaas P. Pruessmann, Markus Weiger, Markus B. Scheidegger, Peter Boesiger. SENSE: Sensitivity encoding for fast MRI. *Magnetic Resonance in Medicine* 1999. 42(5):952-962.

[18] John P. Mugler. Optimized three-dimensional fast-spin-echo MRI. *Journal of Magnetic Resonance Imaging* 2014. 39(4):745-767.

[19] Michael Lustig, David Donoho, John M. Pauly. Sparse MRI: The application of compressed sensing for rapid MR imaging. *Magnetic Resonance in Medicine* 2007. 58(6):1182-1195.

[20] Huanzhou Yu, Scott B. Reeder, Ann Shimakawa, Jean H. Brittain, Norbert J. Pelc. Field map estimation with a region growing scheme for iterative 3-point water-fat decomposition. *Magnetic Resonance in Medicine* 2005. 54(4):1032-1039.

[21] Scott B. Reeder, Zhifei Wen, Huanzhou Yu, Angel R. Pineda, Garry E. Gold, Michael Markl, Norbert J. Pelc. Multicoil Dixon Chemical Species Separation with an Iterative Least-Squares Estimation Method. *Magnetic Resonance in Medicine* 2004. 51(1):35-45.

[22] Dorne, L., Stroman, P., Rolland, C., Auger, M., Alikacem, N., Bronskill, M., Grondin, P., King, M. W., Guidoin, R.. Magnetic resonance study of virgin and explanted silicone breast prostheses. Can proton relaxation times be used to monitor their biostability?. *ASAIO Journal (American Society for Artificial Internal Organs: 1992)* 1994. 40(3):M625–M631.

[23] Daniel M. Krainak, Brain Garra, Sunder S. Rajan. MR Relaxometry of Silicone Breast Implants at 3.0T. In: *Proc Proceedings of the 20th Annual Meeting of the International Society for Magnetic Resonance in Medicine (ISMRM)*; 2012.

[24] Ozturkler, Batu. SMRD: SURE-Based Robust MRI Reconstruction with Diffusion Models. 2023.

[25] Mariya Doneva, Peter Börnert, Holger Eggers, Alfred Mertins, John Pauly, Michael Lustig. Compressed sensing for chemical shift-based water-fat separation. *Magnetic Resonance in Medicine* 2010. 64(6):1749-1759.

[26] Nan Kuei Chen, Alice M. Wyrwicz. Removal of EPI Nyquist ghost artifacts with two-dimensional phase correction. *Magnetic Resonance in Medicine* 2004. 51(6):1247-1253.

[27] Christiane Katharina Kuhl, Sven Klaschik, Peter Mielcarek, Jue Rgen Gieseke, Eva Wardelmann, Hans H Schild. Do T2-Weighted Pulse Sequences Help With the Differential Diagnosis of Enhancing Lesions in Dynamic Breast MRI. *J. Magn. Reson. Imaging* 1999. 9():187-196.

[28] Hernando, Diego, Kellman, Peter, Haldar, Justin P, Liang, Zhi-Pei. Robust water/fat separation in the presence of large field inhomogeneities using a graph cut algorithm. *Magnetic Resonance in Medicine* 2010. 63(1):79–90.

[29] Cui, Chen, Wu, Xiaodong, Newell, John D, Jacob, Mathews. Fat water decomposition using globally optimal surface estimation (GOOSE) algorithm. *Magnetic Resonance in Medicine* 2015. 73(3):1289–1299.

[30] Cui, Chen, Shah, Abhay, Wu, Xiaodong, Jacob, Mathews. A rapid 3D fat–water decomposition method using globally optimal surface estimation (R-GOOSE). *Magnetic Resonance in Medicine* 2018. 79(4):2401–2407.

[31] Borde, Tabea, Wiedemann, Antonia, Stelter, Jonathan, Boehm, Christof, Ruschke, Stefan, Weiss, Kilian, Wu, Mingming, Makowski, Marcus R., Karampinos, Dimitrios C., Fallenberg, Eva M.. Silicone Implant and Fibrous Capsule Assessment Based on water, fat and silicone Images from a Chemical Shift Encoding-Based Species Separation. In: *Proc Proceedings of the 30th Annual Meeting of the International Society for Magnetic Resonance in Medicine (ISMRM)*; 2022.

[32] Dixon, W T. Simple proton spectroscopic imaging. *Radiology* 1984. 153(1):189-194.

[33] Zhou, Xuetong, Daniel, Bruce L., Hargreaves, Brian A., Lee, Philip K.. Distortion-free water-fat separated diffusion-weighted imaging using spatiotemporal joint reconstruction. *Magnetic Resonance in Medicine* 2024. 92(6):2343–2357.

[34] Gorczyca, D. P., Schneider, E., DeBruhl, N. D., Foo, T. K. F., Ahn, C. Y., Sayre, J. W., Shaw, W. W., Bassett, L. W.. Silicone breast implant rupture: Comparison between three-point Dixon and fast spin-echo MR imaging. *American Journal of Roentgenology* 1994. 162(2):305–310.

[35] Hernando, Diego, Liang, Zhi-Pei, Kellman, Peter. Chemical shift-based water/fat separation: a comparison of signal models. *Magnetic Resonance in Medicine* 2010. 64(3):811–822.

[36] Neuro MR Physics group. KS Foundation. .

[37] Boehm, Christian, Diefenbach, Martin N, Makowski, Marcus R, Karampinos, Dimitrios C. Improved body quantitative susceptibility mapping by using a variable-layer single-min-cut graph-cut for field-mapping. *Magnetic Resonance in Medicine* 2021. 85(3):1697–1712.

[38] Gavin Hamilton, Takeshi Yokoo, Mark Bydder, Irene Cruite, Michael E. Schroeder, Claude B. Sirlin, Michael S. Middleton. In vivo characterization of the liver fat 1H MR spectrum. *NMR in Biomedicine* 2011. 24(7):784-790.

[39] Christof Boehm, Maximilian N. Diefenbach, Marcus R. Makowski, Dimitrios C. Karampinos. Improved body quantitative susceptibility mapping by using a variable-layer single-min-cut graph-cut for field-mapping. *Magnetic Resonance in Medicine* 2021. 85(3):1697-1712.

[40] Rebecca Rakow-Penner, Bruce Daniel, Huanzhou Yu, Anne Sawyer-Glover, Gary H. Glover. Relaxation times of breast tissue at 1.5T and 3T measured using IDEAL. *Journal of Magnetic Resonance Imaging* 2006. 23(1):87–91. doi:10.1002/jmri.20469.

[41] Daniel M. Krainak, Brian Garra, Sunder S. Rajan. MR relaxometry of silicone breast implants at 3.0T. *Proceedings of the International Society for Magnetic Resonance in Medicine (ISMRM)* 2012. Abstract 1480.

[42] Diego Hernando, J. Harald Kramer, Scott B. Reeder. Multipeak fat-corrected complex R_2^* relaxometry: Theory, optimization, and clinical validation. *Magnetic Resonance in Medicine* 2013. 70(5):1319–1331. doi:10.1002/mrm.24593.

[43] Daniel Horng, Diego Hernando, Scott B. Reeder. Quantification of liver fat in the presence of iron overload. *Journal of Magnetic Resonance Imaging* 2012. 45(2):428–439. doi:10.1002/jmri.25282.

[44] Peterson, Pernilla, Mansson, Sven. Simultaneous quantification of fat content and fatty acid composition using MR imaging. *Magnetic Resonance in Medicine* 2013. 69(3):688–697. doi:10.1002/mrm.24297.

[45] Takeshi Akasaka, Koichiro Fujimoto, Takashi Yamamoto, Tomohiro Okada, Yoshihisa Fushimi, Atsushi Yamamoto, Takayuki Tanaka, Kouji Togashi. Optimization of regularization parameters in compressed sensing of magnetic resonance angiography: can statistical image metrics mimic radiologists' perception? *PLoS ONE* 2016. 11(1):e0146548.

[46] Kevin F. King, Luca Marinelli, Clifford J. Hardy. Adaptive regularization in compressed sensing using the discrepancy principle. *Proceedings of the International Society for Magnetic Resonance in Medicine (ISMRM)* 2009.

[47] Sujatha Ramani, Thierry Blu, Michael Unser. Monte-Carlo SURE: a black-box optimization of regularization parameters for general denoising algorithms. *IEEE Transactions on Image Processing* 2008. 17(9):1540-1554.

[48] David L. Donoho, Iain M. Johnstone. Adapting to unknown smoothness via wavelet shrinkage. *Journal of the American Statistical Association* 1995. 90(432):1200-1224.

[49] Jin Ren, I. Dimitrov, A. Dean Sherry, Craig R. Malloy. Composition of adipose tissue and marrow fat in humans by ^{1}H NMR at 7 Tesla. *Journal of Lipid Research*, 2008; 49:2055–2062. doi:10.1194/jlr.D800010-JLR200.
